# Prise en charge des corps étrangers enclaves de l’œsophage: à propos de 36 cas

**DOI:** 10.11604/pamj.2017.27.207.7463

**Published:** 2017-07-20

**Authors:** Seydou Togo, Moussa Abdoulaye Ouattara, Xing Li, Shang Wen Yang, Sékou Koumaré

**Affiliations:** 1 Service de Chirurgie Thoracique, CHU Hôpital du Mali, Bamako; 2 23^ème^ Mission d’Aide Médicale Chinoise au Mali; 3 Service de Chirurgie A Hôpital du Point G, Mali

**Keywords:** Œsophage, corps étrangers, enclavés, extraction endoscopique, chirurgie, Esophagus, foreign body, enclaved, endoscopic removal, surgery

## Abstract

L'ingestion de corps étranger de l'œsophage est un motif fréquent de consultation aux urgences pédiatriques. Cependant le phénomène peut se retrouver à tous les âges. Les auteurs décrivent les caractéristiques cliniques, paracliniques et thérapeutiques des corps étrangers enclavés dans l'œsophage pris en charge à l'hôpital du Mali. Il s'agit d'une étude prospective, menée entre janvier 2011 et décembre 2014 incluant tous les cas d'ingestion de corps étrangers enclavés dans l'œsophage. Au total 36 patients ont été pris en charge par des moyens endoscopiques ou chirurgicaux. L'âge moyen était de 6 ans (extrêmes: 14 mois- 62 ans). Le sexe masculin était dominant avec un sexe ratio de 1,75. Les corps étrangers étaient bloqués dans le rétrécissement crico-pharyngien dans 69,45% des cas suivi du rétrécissement aortique dans 22,22% des cas. Le délai d'extraction du corps étranger en moyenne était de 7 heures 30. La fibroscopie rigide a permis l'extraction du corps étranger dans 88,89% des cas. Une chirurgie par thoracotomie a permis d'extraire le corps étranger dans 5,55%. Les corps étrangers de l'œsophage peuvent se retrouver à tout âge mais restent plus fréquent chez l'enfant. L'extraction endoscopique est la man'uvre la plus réalisée mais la chirurgie pour extraction d'un corps étranger bloqué dans l'œsophage bien que rare reste le dernier recours à cause souvent de leur nature et de la survenue des complications. Le meilleur moyen pour lutter contre ces accidents reste la prévention.

## Abstract (en)

Esophageal foreign bodies are a frequent reason for consultation in the Pediatric Emergency Department. However, they can occur at all ages. This study aims to highlight the clinical, paraclinical and therapeutic features of esophageal foreign bodies management at the Hospital in Mali. We conducted a prospective study of all cases of ingestion of foreign bodies between January 2011 and December 2014. A total of 36 patients underwent endoscopic or surgical treatment. The average age was 6 years (with a range from 14 months to 62 years). They mainly affected male patients with a sex ratio of 1.75. Foreign bodies were blocked in the cricopharyngeal shrinkage in 69.45% of cases, 22.22% of whom had subsequent aortic shrinkage. The average time of foreign body removal was 7.30 hours. Rigid fibroscopy allowed the removal of the foreign body in 88.89% of cases. Thoracotomy allowed the removal of the foreign body in 5.55%. Esophageal foreign bodies can occur at all ages but they are more frequent among children. Endoscopic removal is the gold standard treatment but surgical removal of a blocked esophageal foreign body, although rare, is the last resort, due to the nature of the foreign body and to the occurrence of complications. The best way to reduce accidents is prevention.

## Introduction

L'ingestion de corps étranger est un motif fréquent de consultation aux urgences pédiatriques, cependant le phénomène peut se retrouver à tous les âges [1]. Chez l'adulte les auteurs affirment que la prothèse dentaire est le facteur favorisant du fait de l'absence de contact alimentaire avec la muqueuse du palais [2]. Les habitudes alimentaires conditionnent certains types de corps étrangers ainsi que l'âge des patients car les enfants en phase orale sont à risque [3]. Bien que le développement de l'endoscopie ait permis de diminuer considérablement la mortalité, les corps étrangers ingérés restent source de morbidité dans les pays sous développés liée aux moyens diagnostiques souvent inaccessibles et du fait de leur fréquence [4]. Le but de ce travail est de décrire les caractéristiques cliniques, paracliniques et thérapeutiques à mi-parcours des corps étrangers enclavés dans l'œsophage pris en charge à l'hôpital du Mali et faire une revue de la littérature.

## Méthodes

**Figure 1:**
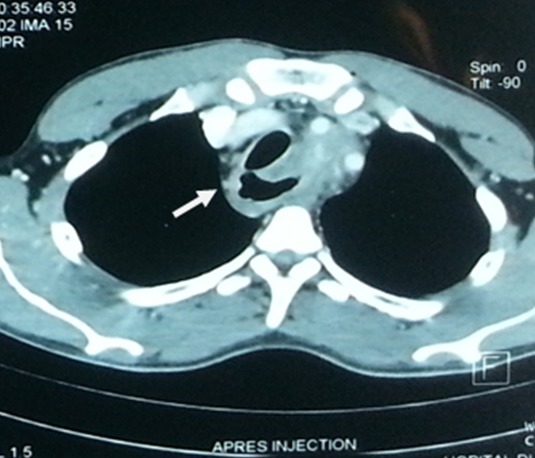
TDM thoracique montrant un corps étranger non radio opaque, pointu incarcéré dans le muscle œsophagien

Il s'agit d'une étude prospective, menée entre janvier 2011 et décembre 2014 à l'hôpital du Mali, incluant tous les cas d'ingestion de corps étrangers enclavés dans l'œsophage, hospitalisés dans les services des urgences, de chirurgie thoracique, de pédiatrie et également ceux reçu en consultation externe qui ont été pris en charge. Au total nous avons enregistré 36 cas (22 hommes et 14 femmes) d'ingestion de corps étrangers enclavés dans l'œsophage pendant la période. Etaient inclus dans cette étude tous les cas de corps étrangers ingérés et qui se situait dans la lumière de l'œsophage lors d'un contrôle radiologique ou endoscopique au moment de leur admission. Etaient exclu de l'étude tous les cas d'ingestion de corps étrangers se situant dans d'autres parties du tube digestif en dehors de l'œsophage. La radiographie thoracique a été réalisée chez l'ensemble des patients et la fibroscopie chez 35 patients. Un cas a nécessité un examen supplémentaire de tomodensitométrie thoracique lorsque le corps étranger était inextirpable par les moyens endoscopiques (Figure 1). Le transit oeso-gastroduodénal (TOGD) a été réalisé en absence de notion d'ingestion de corps étranger chez un patient immunodéprimé et qui présentait des vomissements post prandiaux précoces. Les données comprenant l'âge, le sexe, la symptomatologie, le délai de consultation et d'extraction, la localisation, la nature des corps étrangers, l'extraction et la morbi-mortalité liée à la prise en charge ont été étudiées. Les résultats sont exprimés en pourcentage pour les différentes valeurs exprimées.

## Résultats

**Répartition des cas selon l'âge, le sexe et la symptomatologie**: 83.33% des patients (n=30) étaient des enfants avec un âge inferieur à 13 ans et 16.67% (n=6) étaient des adultes (âge entre 18 et 62 ans). L'âge moyen était de 6 ans (extrêmes: 14 mois- 62 ans) avec un écart type de 8,51. Le sexe masculin était dominant avec un sexe ratio de 1,75. A l'admission 97.22%(n=35) patients étaient symptomatiques. Les symptômes retrouvés chez les patients lors de l'admission sont représentés dans le Tableau 1.

**Tableau 1:** Caractéristiques cliniques et paracliniques des patients

Signes cliniques	Nbre patients (n=36)	Pourcentage(%)
Vomissement	16	44,44
Douleur retro-sternale	11	30,55
Dysphagie	7	19,44
Toux	6	16,66
Hypersialorrhée	3	8,33
dyspnée	2	5,55
Agitation	2	5,55
**Moyens diagnostique**	**Nbre**	**(%)**
RX thorax	36	100
Fibroscopie	35	97,22
TOGD	1	2,77
TDM thoracique	1	2,77
**Site de blocage**	**Nbre**	**%**
Muscle crico-pharyngien	25	69,45
Crosse aortique	8	22,22
Sphincter inf, œsophage	3	8,33

**Figure 2:**
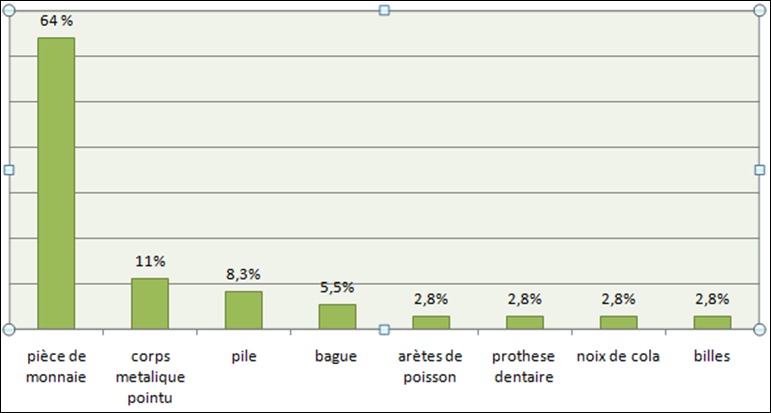
Répartition des corps étrangers ingérés en fonction de leurs natures

**Figure 3:**
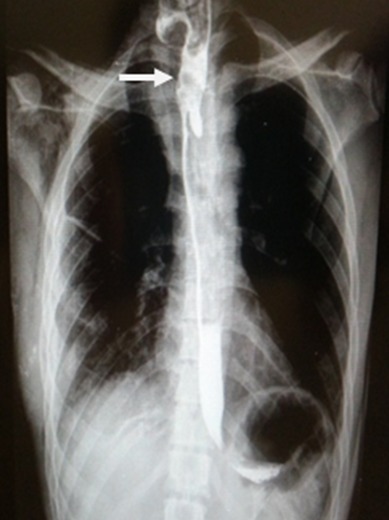
TOGD montrant une image lacunaire de corps étranger non radio opaque dans l’œsophage supérieur

**Localisation et nature du corps étranger**: Les corps étrangers étaient bloqués dans le rétrécissement crico-pharyngien dans 69.45% des cas suivi du rétrécissement de la crosse aortique dans 22,22% (Tableau 1). Selon la nature des corps étrangers ingérés les pièces de monnaie étaient les plus représentés (64%) suivi des objets métalliques pointus (11%) (Figure 2). La radiographie a permis de mettre en évidence le corps étranger dans 80.55% (n= 29) des cas. La fibroscopie a permis de localiser le corps étranger dans 35 cas (97,22%). Ces corps étrangers étaient radio-opaque ou non. Le TOGD a permis de localiser un cas de corps étranger non radio-opaque. (Présence d'image de lacune) (Figure 3).

**Extraction du corps étranger**: Le délai moyen de consultation en urgence est de 12 heures et d'extraction du corps étranger, de 7 heures 30. La fibroscopie digestive haute à l'aide du fibroscope rigide a permis l'extraction du corps étranger dans 88,89% des cas (n=32). L'élimination spontanée a été obtenue au bout de 72 h dans 5,55% des cas (n=2). La chirurgie d'œsophagotomie a permis d'extraire le corps étranger (après échec de plusieurs tentatives endoscopiques) dans 2,77% (n=1). Il s'agissait d'un patient âgé de 42 ans qui a ingéré sa prothèse dentaire de façon accidentelle et qui depuis 10 ans était suivi dans les structures de santé périphérique pour œsophagite. Une thoracotomie a été réalisée suite à une fistule 'sophagienne dans 2,77% des cas (n= 1) qui a permis d'extraire le corps étranger et aussi de fermer la fistule par un lambeau de muscle intercostal prélevé. Il s'agissait d'un corps métallique (ressort) à extrémité pointue, enclavé qui a perforé l'œsophage dans sa partie medio-thoracique en sous aortique. Au total deux cas (5,55%) ont nécessité une intervention chirurgicale. Les suites opératoires étaient simples.

**Evolution**: L'évolution était favorable chez l'ensemble des patients après extraction du corps étranger. Les complications représentaient 5,56%; Les complications étaient liées à la lésion de la sphère buccale survenant de facon iatrogène lors des tentatives d'extraction avant la consultation et aussi l'infection liée à la perforation œsophagienne par le corps étranger pointu. La mortalité était nulle dans notre étude.

## Discussion

La population des patients ingérant des corps étrangers est du reste à 80% pédiatrique avec un pic de fréquence entre 6 mois et 3 ans (70%) contre 30% entre 3 et 12 ans [2, 3, 5]. Les alcooliques sont des patients à risques ainsi que ceux présentant des antécédents de malformations ou de chirurgie digestive; il en est de même pour des adultes édentés, âgés [2, 4, 5]. Dans notre étude, la population ayant un âge inferieur à 13 ans représentait 83,33% contre 16,67% d'adulte. Cela confirme bien le risque très élevé d'ingestion de corps étranger chez les enfants surtout lorsqu'ils sont en phase orale. Dans la plupart des cas, il est possible de faire le diagnostic d'ingestion d'un corps étranger à l'interrogatoire mais dans certains cas, cela n'est pas évident et il faut savoir interroger l'entourage ou les témoins, car il existe de fréquentes formes inaperçues d'ingestion de corps étrangers [4, 6]. Le site du blocage s'effectue le plus souvent au niveau de la glotte, des valécules, du larynx, du muscle crico-pharyngien, de la crosse aortique et du sphincter inférieur de l'œsophage [5, 6]. Selon les auteurs dans 60 à 80% des cas, le blocage se situe au niveau du muscle crico-pharyngien, 10 à 20% au niveau de la crosse aortique et dans 5% à 20% au niveau du sphincter inférieur de l'œsophage [3, 5, 6]. Ces résultats concordent avec notre étude qui a retrouvé le blocage au niveau de muscle crico-pharyngien dans 68,45%, de la crosse aortique dans 22,22% et du sphincter inférieur 'sophagien dans 8,33%. Ces sites sont les zones de rétrécissement anatomique de l'œsophage. Les corps étrangers situés dans les autres sites supérieurs tels que la glotte, le larynx sont généralement pris en charge par les services d'otorhinolaryngologie et n'ont pas fait l'objet de notre étude. L'ingestion est symptomatique dans un grand nombre de cas surtout lorsque le corps étranger reste bloqué dans l'œsophage (syndrome obstructif) pendant un long temps ou lorsqu'il s'agit de corps étrangers long ou souvent pointus qui perfore l'œsophage [7]. La prise en charge dépend du type de l'objet ingéré et de sa localisation. Les objets tranchants correspondent à une urgence s'ils sont impactés au niveau de l'œsophage en raison des risques de perforation ou hémorragiques [8]. Les objets longs (supérieurs à 6 cm) et les gros objets «mousses» (blunt) qui créent un syndrome obstructif nécessitent une extraction en urgence [5, 6, 9]. Dans notre étude, parmi les cas d'objets pointus retirés deux cas ont nécessité une intervention chirurgicale parmi lesquelles un cas pour perforation de l'œsophage et également un second cas pour corps étranger enclavé pendant 10 ans après échec de plusieurs tentatives d'extraction endoscopique. Ces cas ont été pris en charge comme une urgence différée compte tenu le plus souvent du retard diagnostique car certains de nos patients étaient en provenance des zones rurales où les centres de santé sont limités par rapport aux moyens diagnostiques et très souvent le plateau technique est absent. Cependant plusieurs autres cas ont fait l'objet de prise en charge en urgence. La plupart de ces patients sont en provenance de milieu urbain.

Quatre-vingt à 90% des corps étrangers ingérés passent spontanément, 10 à 20% nécessitent des man'uvres non chirurgicales d'extraction et moins d'1% le recours à la chirurgie [4, 10]. Dans l'étude la fibroscopie rigide a été le moyen le plus utilisé pour l'extraction des corps étrangers enclavés. Cependant certains corps étrangers ronds enclavés ont pu évoluer dans l'estomac et être éliminés. Cela a pu s'observer lorsque les patients ont été consultés très tôt avec un examen radiologique initiale de diagnostique réalisé dans les structures périphériques et lors du second contrôle radiologique dans notre hôpital il ya eu une évolution dans la progression du corps étranger mais toujours étant situé dans l'œsophage. De tels cas doivent attirer notre attention et doivent être observés en dehors de tout risque de complications potentielles. Ainsi pour les objets ronds bloqués au niveau de l'œsophage, il faut savoir attendre 24 heures en l'absence de syndrome obstructif. La majorité des corps étrangers ingérés sont radio-opaques, visibles sur une radiographie du thorax élargie au cou et à la cavité gastrique [11]. Au niveau du cou et du thorax, le cliché de profil confirme si nécessaire la position postérieure du corps étranger œsophagien par rapport aux clartés antérieures du larynx, de la trachée et de la carène. Les corps étrangers métalliques ou en verre sont le plus souvent radio-opaques; il s'agit de pièces, piles, aiguilles, épingles. Par contre, les cartilages, les os, les arêtes, les morceaux de plastique, parfois verre ou alliage ne sont pas toujours radio opaques à la radiographie [4, 5, 8, 12]. Les corps étrangers alimentaires sont le plus souvent radio-transparents. Il faut rapidement réaliser des radiographies standards complètes, bi-plans surtout s'il y a un doute entre inhalation (arbre respiratoire) et ingestion (tractus digestif). Comme le confirme plusieurs auteurs, à la radiographie pulmonaire, une pièce, ronde de face et linéaire de profil est le plus souvent dans l'œsophage [2, 9, 10, 12].

Le recours à d'autres examens d'imagerie pour localiser le corps étranger (échographie, TOGD, tomodensitométrie, IRM) n'est habituellement pas nécessaire. Lorsque le corps étranger est radio-transparent, une endoscopie digestive haute permet de confirmer sa présence dans l'œsophage. Pour les corps radio transparents dans l'étude, l'endoscopie a toujours été réalisée d'emblé pour poser le diagnostique et pendant le même temps l'extraction du corps étranger était réalisée par des manœuvres très douces afin d'éviter les lésions secondaires liées souvent à la nature des corps étrangers. La manœuvre doit être abandonnée après 2 ou 3 tentatives d'extraction pour recourir à d'autre examen complémentaire tel que le scanner afin d'éviter le risque de lésion iatrogène [9, 12]. Chez un de nos patients, au total 7 tentatives d'extractions endoscopique réalisées dans 3 différents hôpitaux ont été sans succès et la manœuvre fut abandonnée pour réaliser le scanner afin de comprendre le mécanisme de blocage ou les complications et permettre de prendre une décision chirurgicale. La prothèse était incarcérée dans le muscle œsophagien et il existait tout autour une fibrose qui empêchait son extraction par les moyens endoscopiques. En cas de doute sur la présence d'un corps étranger œsophagien non radio-opaque, une opacification de l'œsophage avec un produit de contraste hydrosoluble peut être réalisée pour visualiser un corps étranger œsophagien non radio-transparent [9, 13]. Dans notre étude l'opacification de l'œsophage par l'examen du transit œsogastroduodénal nous a permis de suspecter la présence d'un corps étranger radio-transparent chez un patient immunodéprimé qui était traité pour œsophagite mycosique et a été pris en charge. Ces patients sont le plus souvent stigmatisés dans les centres non spécialisés et ne bénéficient pas le plus souvent d'examen exhaustif endoscopique. Si le corps étranger n'est pas visualisé par les moyens endoscopiques, la possibilité d'ingestion d'un corps étranger radio-transparent ou non ne peut pas être écartée, car il peut avoir migré dans le tractus digestif inférieur ou souvent perforer l'œsophage et passer à travers [9, 10, 13]. Des examens complémentaires plus exhaustifs peuvent être nécessaire pour poser souvent le diagnostique. Les complications des corps étrangers de l'œsophage peuvent être graves comme les perforations et les infections graves (médiastinite, pneumopathie) [14]. Bien que dans notre étude le taux de complication reste faible, il peut atteindre jusqu'à 20%. La survenue des complications dépendent aussi de la coopération du patient, l'expérience du médecin, la nature du corps, la localisation, le délai de prise en charge et la disponibilité du plateau technique.

## Conclusion

Les corps étrangers de l'œsophage peuvent se retrouver à tout âge mais restent plus fréquent chez l'enfant. La majorité des corps étrangers sont ingérés accidentellement. L'extraction endoscopique est la manœuvre la plus souvent réalisée mais la chirurgie pour extraction d'un corps étranger bloqué dans le tube digestif bien que rare reste le dernier recours à cause souvent de leur nature et de la survenue des complications. Le meilleur moyen pour lutter contre ces accidents reste la prévention.

### Etat des connaissances actuelles sur le sujet

- Les corps étrangers de l'œsophage sont des pathologies fréquentes en milieu hospitalier. L'extraction est courante par endoscopie rigide. La morbi-mortalité est élevée en cas de complications.

### Contribution de notre étude à la connaissance

- Nécessité de realization de la Tomodensitométrie Thoracique pour comprendre le mécanisme de blocage en cas d'impossibilité d'extraction endoscopique. Le transit oesogastro-duodénal peut permettre d'identifier des corps étrangers non radio opaques en milieu peu équipé ou chez des patients stigmatisés (ex. VIH positif). La chirurgie pour extraction du corps étranger doit être le dernier recours dans la prise en charge.

## Conflits d’intérêts

Les auteurs ne déclarent aucun conflit d'intérêt.
